# Efficacy and validation of a clinical model to predict acute kidney injury in severe pneumonia requiring mechanical ventilation in elderly patients: a multicenter retrospective observational analysis

**DOI:** 10.3389/fmed.2025.1685110

**Published:** 2025-12-05

**Authors:** Li Yao, Wenjing Ding, Jingjing Zhao, Xiang Fang, Kunlun Niu, Di Ma, Ting Chen, Jingyu Li, Yu Fu, Yuan Zhan, Gaoqiang Ling, Wei Wang

**Affiliations:** 1 Intensive Care Unit, The Second People’s Hospital of Hefei, Hefei Hospital Affiliated to Anhui Medical University, Hefei, China; 2 The Second Clinical Medical School, Anhui University of Chinese Medicine, Hefei, China; 3 Department of Emergency, Intensive Care Unit, The First Affiliated Hospital of Anhui Medical University, Hefei, China; 4 Intensive Care Unit, First Affiliated Hospital of Anhui University of Chinese Medicine, Hefei, China; 5 Institute of Artificial Intelligence, Hefei Comprehensive National Science Center, Hefei, China; 6 School of Medicine, Xian Jiaotong University, Xi’an, China; 7 Department of Colorectum, First Affiliated Hospital of Anhui University of Chinese Medicine, Hefei, China

**Keywords:** severe pneumonia requiring mechanical ventilation, acute kidney injury, predictive model, risk factors, nomogram

## Abstract

**Background:**

The objective of our retrospective multicenter analysis was to identify risk factors and construct a statistical model for predicting acute kidney injury (AKI) among elderly patients with severe pneumonia requiring mechanical ventilation (SPRMV) in elderly patients in different intensive care units (ICU).

**Methods:**

We aimed to utilize a multi-center retrospective analysis, including 353 cases of SPRMV patients diagnosed and treated in the ICU of the Hefei Second People’s Hospital between May 2018 and February 2025 as a training dataset, and 151 participants were admitted to the ICU of the First Affiliated Hospital of Anhui Medical University between June 2020 and March 2025, considered as a validation dataset. Both univariate and multivariate logistic regression analyses were utilized to investigate the risk factors of SPRMV with AKI. After that, our predictive model was evaluated using a nomogram, a receiver operating characteristic (ROC) curve for discrimination, calibration curves, and decision curve analysis (DCA) curves for clinical validity.

**Results:**

Our multivariate logistic regression analysis indicated that CREA, SOFA, APACHE II, driving pressure, mechanical kinetic energy, CRP/ALB, and MAP are independent risk factors of SPRMV in the elderly patients with AKI. A nomogram of SPRMV in elderly patients with AKI was constructed. The ROC curve revealed that our predictive model showed good predictive efficacy with an area under curve (AUC) of 0.920 (95% confidence intervals (CI) = 0.892–0.948) with a specificity of 0.993 and sensitivity of 0.763 in the training dataset and an AUC value of 0.938 (95%CI = 0.899–0.977) with a specificity of 0.952 and sensitivity of 0.854 in the validation dataset. Moreover, calibration and DCA curves demonstrated that our predictive model had a good fit, better net benefit, and higher predictive efficiency for SPRMV in the elderly patients with AKI.

**Conclusion:**

Our predictive model demonstrated that CREA, SOFA, APACHE II, driving pressure, mechanical kinetic energy, CRP/ALB, and MAP were the independent risk factors of AKI in SPRMV in the elderly patients with high accuracy and good calibration.

## Introduction

Severe pneumonia is a serious infectious disease that is common among the elderly and has a high mortality rate (1–7). Mechanical ventilation, as a key life-supporting therapy, is widely used in the treatment of patients with severe pneumonia (8). However, the incidence of acute kidney injury (AKI) in mechanically ventilated patients remains relatively high, which has become an important determinant affecting the prognosis and quality of life of patients (9). AKI not only complicates the treatment process, significantly prolongs hospitalization time, and increases medical costs but also significantly increases the risk of death for patients (10). The risk of AKI during mechanical ventilation is further increased in elderly patients due to weakened immune function, diminished organ reserves, and various underlying diseases (11). At present, there is a validated lack of validated risk-prediction tools for AKI in elderly patients with severe pneumonia undergoing mechanical ventilation, and there is no systematic validation based on large amounts of clinical data from multiple centers, which to some extent limits the accurate identification and early intervention of high-risk patients in clinical practice.

The pathogenesis of AKI is extremely complex in patients with severe pneumonia undergoing mechanical ventilation. Infection and systemic inflammatory response trigger the release of a large amount of inflammatory mediators (12), causing direct damage to renal tubular epithelial cells, inducing microcirculation disorders and cell apoptosis (13, 14). During mechanical ventilation, increased positive chest pressure leads to a decrease in venous return and a decrease in renal blood flow perfusion, which exacerbates renal ischemia and hypoxia (15). Furthermore, common factors such as acid–base imbalance, electrolyte imbalance, and metabolic disorders in severe conditions also significantly increase the burden on the kidneys, promoting the occurrence and development of AKI (16). These pathological processes interact with each other to form a vicious cycle, resulting in a poor prognosis for elderly critically ill patients, which is extremely poor once AKI occurs (17, 18). Specifically, we note that the majority of previous AKI prediction models were constructed for broad-based intensive care units (ICU) populations and did not specifically target elderly patients with severe pneumonia requiring mechanical ventilation (SPRMV). Furthermore, most modeling studies failed to perform any external validation and, in addition, did not consider either ventilation-related or inflammatory parameters relevant to this population. To fill these specific gaps, our study combines clinical, inflammatory, and mechanical ventilation variables from multiple centers to establish and validate a model predictive of AKI that is more directly targeted and better generalizable.

In view of this, this study retrospectively analyzed clinical data from elderly patients with severe pneumonia undergoing mechanical ventilation admitted to multiple centers by comprehensively evaluating infection- and inflammation-related markers, ventilation parameters, underlying diseases, and medication use. A rigorous multivariate statistical method was used to establish and validate an AKI risk prediction model. This model aims to provide a reliable and clinically applicable tool for clinical practice, enabling early prediction and precise management of high-risk patients, promoting the development of individualized treatment plans, and ultimately improving the treatment effectiveness and quality of life of patients.

## Materials and methods

### Study population and analysis design

We retrospectively collected individuals diagnosed with SPRMV from two multicenter ICU, with a total of 504 SPRMV individuals, of which a total of 353 participants were admitted to the ICU of the Hefei Second People’s Hospital between May 2018 and February 2025 and considered as a training dataset. A total of 151 participants were admitted to the ICU of the First Affiliated Hospital of Anhui Medical University between June 2020 and March 2025 and considered as a validation dataset. Our analysis was approved by the Ethics Committee of both the Hefei Second People’s Hospital and the First Affiliated Hospital of Anhui Medical University, and the study followed the Declaration of Helsinki.

In our study, AKI was defined according to the Kidney Disease: Improving Global Outcomes (KDIGO) criteria, which include an increase in serum creatinine by ≥ 0.3 mg/dL within 48 h, or an increase to ≥ 1.5 times the baseline within the prior 7 days, or a urine output of < 0.5 mL/kg/h for 6 h. The inclusive and exclusive criteria for our SPRMV patients’ clinical data collection are as follows: Inclusive criteria: (a) elderly patients aged ≥ 65 years old; (b) diagnosed with SPRMV during hospitalization in two ICUs; (c) all patients diagnosed with ARI undergo renal ultrasound examination to rule out posterior renal factors such as ureteral obstruction. The exclusive criteria are as follows: (a) age less than 65 years old; (b) chronic renal disease stage 4–5 (eGFR (glomerular filtration rate) < 30 mL/min/1.73 m^2^) or previous ≥ 4 weeks of blood/peritoneal dialysis; (c) those who die within 48 h of admission, voluntarily give up treatment, have family members request discharge, or have advanced malignant tumors and receive palliative treatment, with an expected survival period of less than 3 months; (d) individuals with incomplete medical history data.

### Data definition

#### Social-demographic characteristics

We recorded case history information on four social-demographic characteristics, including gender, age, education level, and marital status. Gender was categorized as male or female. The education level was defined as less than primary school, middle school, or upper college. Marital status was defined as married, unmarried, or divorced.

#### Clinicopathologic characteristics

The following clinicopathologic characteristics were included in our present analysis: drinking consumption (yes, no), smoking consumption (yes, no), daily diet habit (regular, irregular), depression (yes, no), cardiovascular disease (CVD) (yes, no), hypertension (yes, no), diabetes (yes, no), coronary heart disease (CHD) (yes, no), hemoglobin (HGB), red blood cell (RBC) (1,012/L), white blood cell (WBC) (109/L), creatinine (CREA) (μmol/L), lactic acid (LAC) (mmol/L), albumin (ALB) (g/L), total bilirubin (TBIL) (μmol/L), alanine aminotransferase (ALT) (U/L), aspartate aminotransferase (AST) (U/L), procalcitonin (PCT) (ng/L), mechanical ventilation time (h), duration of antibiotic use (h), length of stay in ICU (day), driving pressure (mmHg), mechanical kinetic energy (J), CRP/ALB and mean arterial pressure (MAP) (mmHg), sequential organ failure assessment (SOFA) (19) and acute physiology and chronic health evaluation II (APACHE II) (20), of which mechanical kinetic energy, MAP, SOFA and APACHE II are the worst value within 24 h of admission. To ensure data accuracy and completeness, we used double-checking procedures.

In this study, a dual confirmation process was used for the diagnosis of depressive disorders. First, use the Zung Self-Rating Depression Scale (SDS) for screening (a standard score of ≥ 50 indicates a positive screening result) (21). Subsequently, all screened positive individuals were independently clinically evaluated by specialized physicians in the hospital’s psychological outpatient department. The final diagnosis is made only when both the screening results of the equivalent scale and the clinical judgment of the specialist support a depressive disorder. The basis for determining type 2 diabetes in this study is meeting the following two criteria. First, the patient must have an admission glycated hemoglobin (HbA1c) ≥ 6.5%. Second, a type 2 diabetes diagnosis clearly recorded in the admission history: (a) Must have a clear diagnosis made by a specialist endocrinologist from a second- or higher-level hospital in the past; (b) The medical history should include specific diagnosis dates, treatment plans (such as records of oral hypoglycemic drugs/insulin use), or previous blood glucose monitoring results.

The mechanical kinetic energy (MKE) was the ventilator-derived energy delivered to the respiratory system each minute, with conceptual grounding in the mechanical power theory, as described in (22). It was therefore possible to introduce a valid, though simplified, approximation, enhancing its clinical feasibility (23):


MKE=0.098×RR×VT×[Ppeak−0.5×(Pplat−PEEP)]

Where RR is respiratory rate (unit: breaths/min); VT is tidal volume (unit: L); Ppeak is peak inspiratory pressure (unit: cmH₂O); Pplat is plateau pressure (unit: cmH₂O); and PEEP is positive end-expiratory pressure (unit: cmH₂O). The value 0.098 is a conversion constant from cmH₂O·L/min to joules per minute (J/min). This approximation has been shown to correlate well with the complete mechanical power model and represents the total mechanical energy load applied to the lung.

### Statistical analysis

The association between the incidence rate of SPRMV and 32 potential risk factors or related diseases was examined using R software. For continuous clinical variables, baseline is expressed as the mean (standard deviation), and the *p*-value is calculated using a t-test. Furthermore, categorical variables are displayed as numbers (*N*) or proportions (%). For large samples (*N* ≥ 40), the chi-square test is chosen as the testing method; otherwise, Fisher’s exact test is used. Afterwards, univariate logistic regression analysis was used to analyze the recorded clinical data to control for confounding factors. We further utilize multiple logistic regression analysis to explore the association between the obtained independent risk factors and SPRMV. Besides, we conducted a correlation analysis of independent features using the function “cor()” and obtained a heatmap of the correlation matrix, which displays a graphical representation of the strength and direction of relationships between features in a dataset using color coding. According to the Pearson method, the correlation coefficient displayed in each cell of the matrix ranges from −1 to 1 (24). Furthermore, an advanced nomogram was developed to illustrate the risk of AKI in SPRMV elderly patients.

The discrimination of our predictive model is evaluated using receiver operating characteristic (ROC) curve analysis; the larger the area under the ROC (AUC), the better the discrimination ability. To evaluate the accuracy and clinical effectiveness of our regression model, we utilized a calibration curve and decision curve analysis (DCA).

All the establishment of clinical predictive model and analysis were used with R version 4.4.2 (http://www.r-project.org, R Foundation for Statistical Computing). For baseline characteristics, we utilized the “tableone” package and obtained the table based on both the “flextable” and “officer” packages. To establish the linear regression model, both the “rms” and “regplot” packages were used to plot the advanced nomogram for subsequent analysis. Furthermore, we plotted ROC and DCA curves based on the “pROC” and “rmda” packages, respectively. A two-bilateral *p* value less than 0.05 was considered statistically significant.

## Results

### Participant baseline characteristics

In the current study, detailed baseline information of the socio-demographic and clinicopathologic characteristics of both the training and validation datasets is presented in Table 1. We recorded 504 SPRMV patients across two hospitals, including 300 AKI and 204 non-AKI patients, as shown in Supplementary Table S1. For the training dataset, there were a total of 353 SPRMV patients with non-AKI (*N* = 142, 40.2%) and AKI (*N* = 211, 59.8%), consisting of 223 male patients (63.2%) and 130 female patients (36.8%), and the mean (SD) age was 80.92 ± 7.54 in AKI patients and 81.23 ± 7.69 in non-AKI patients. For the validation dataset, with a total of 151 SPRMV patients with non-AKI (*N* = 62, 41.1%) and AKI (*N* = 89, 58.9%), with 90 male patients (59.6%) and 61 female patients (40.4%), the mean (SD) age was 80.33 ± 7.80 in AKI patients and 81.60 ± 7.99 in non-AKI patients, as shown in Table 1 and Supplementary Table S2.

**Table 1:** Baseline characteristics of SPRMV elderly patients based on training dataset and validation dataset.

Characteristics	Training dataset (*N* = 353)	Validation dataset (*N* = 151)	*p*-value
Gender			0.511
Male	223 (63.2%)	90 (59.6%)	
Female	130 (36.8%)	61 (40.4%)	
Age, mean (SD), years	81.05 ± 7.59	80.85 ± 7.88	0.791
Education			0.759
Less than primary school	122 (34.6%)	48 (31.8%)	
Middle school	113 (32.0%)	53 (35.1%)	
Upper college	118 (33.4%)	50 (33.1%)	
Marital status			0.995
Married	126 (35.7%)	54 (35.8%)	
Unmarried	130 (36.8%)	55 (36.4%)	
Divorced	97 (27.5%)	42 (27.8%)	
Drinking consumption			0.361
Yes	165 (46.7%)	78 (51.7%)	
No	188 (53.3%)	73 (48.3%)	
Smoking consumption			0.098
Yes	182 (51.6%)	65 (43.0%)	
No	171 (48.4%)	86 (57.0%)	
Diet			0.893
Regular	191 (54.1%)	80 (53.0%)	
Irregular	162 (45.9%)	71 (47.0%)	
Depression			0.472
Yes	166 (47.0%)	77 (51.0%)	
No	187 (53.0%)	74 (49.0%)	
CVD			0.969
Yes	180 (51.0%)	76 (50.3%)	
No	173 (49.0%)	75 (49.7%)	
Hypertension			0.509
Yes	167 (47.3%)	77 (51.0%)	
No	186 (52.7%)	74 (49.0%)	
Diabetes			0.402
Yes	175 (49.6%)	68 (45.0%)	
No	178 (50.4%)	83 (55.0%)	
CHD			0.381
Yes	175 (49.6%)	82 (54.3%)	
No	178 (50.4%)	69 (45.7%)	
HGB, mean (SD), g/L	130.16 ± 10.67	128.07 ± 11.01	0.047
RBC, mean (SD), 10^12^/L	4.45 ± 0.37	4.48 ± 0.36	0.38
WBC, mean (SD), 10^9^/L	6.85 ± 1.58	6.64 ± 1.72	0.172
CREA, mean (SD), μmol/L	170.99 ± 102.97	162.72 ± 99.01	0.404
LAC, mean (SD), mmol/L	1.50 ± 0.40	1.51 ± 0.38	0.651
ALB, mean (SD), g/L	1.41 ± 0.41	1.33 ± 0.41	0.061
TBIL, mean (SD), μmol/L	12.07 ± 4.73	12.58 ± 4.52	0.262
ALT, mean (SD), U/L	23.51 ± 9.31	24.90 ± 9.24	0.124
AST, mean (SD), U/L	26.08 ± 5.38	26.13 ± 5.76	0.921
PCT, mean (SD), ng/L	3.75 ± 8.68	5.30 ± 10.32	0.084
Mechanical ventilation time, mean (SD), h	206.55 ± 116.44	205.74 ± 119.12	0.944
Duration of antibiotic use, mean (SD), h	18.76 ± 10.64	18.22 ± 11.38	0.605
Length of stay in ICU, mean (SD), days	19.01 ± 10.81	18.44 ± 11.52	0.596
SOFA, mean (SD)	13.61 ± 5.34	13.03 ± 5.54	0.271
APACHE II, mean (SD)	17.40 ± 7.16	18.03 ± 6.97	0.36
Driving pressure, mean (SD), mmHg	18.48 ± 8.87	19.55 ± 8.59	0.21
Mechanical kinetic energy, mean (SD), J	26.28 ± 10.42	25.93 ± 10.61	0.734
CRP/ALB, mean (SD)	1.53 ± 0.58	1.61 ± 0.60	0.174
MAP, mean (SD), mmHg	103.88 ± 18.16	105.17 ± 18.39	0.467
AKI			0.94
Yes	211 (59.8%)	89 (58.9%)	
No	142 (40.2%)	62 (41.1%)	

To present the model development steps clearly, we calculated all characteristics and their variance inflation factors (VIFs) to address multicollinearity, as shown in Supplementary Tables S3, S4. Since the VIF values for the characteristics are all less than 10, except for duration of antibiotic use and length of stay in ICU. As shown in the following text, these two characteristics did not participate in the construction of the clinical model in this article. Hence, there is no multicollinearity problem in the subsequent model construction.

### Correlation heatmap of predictive characteristics in the training dataset

**Figure 1:**
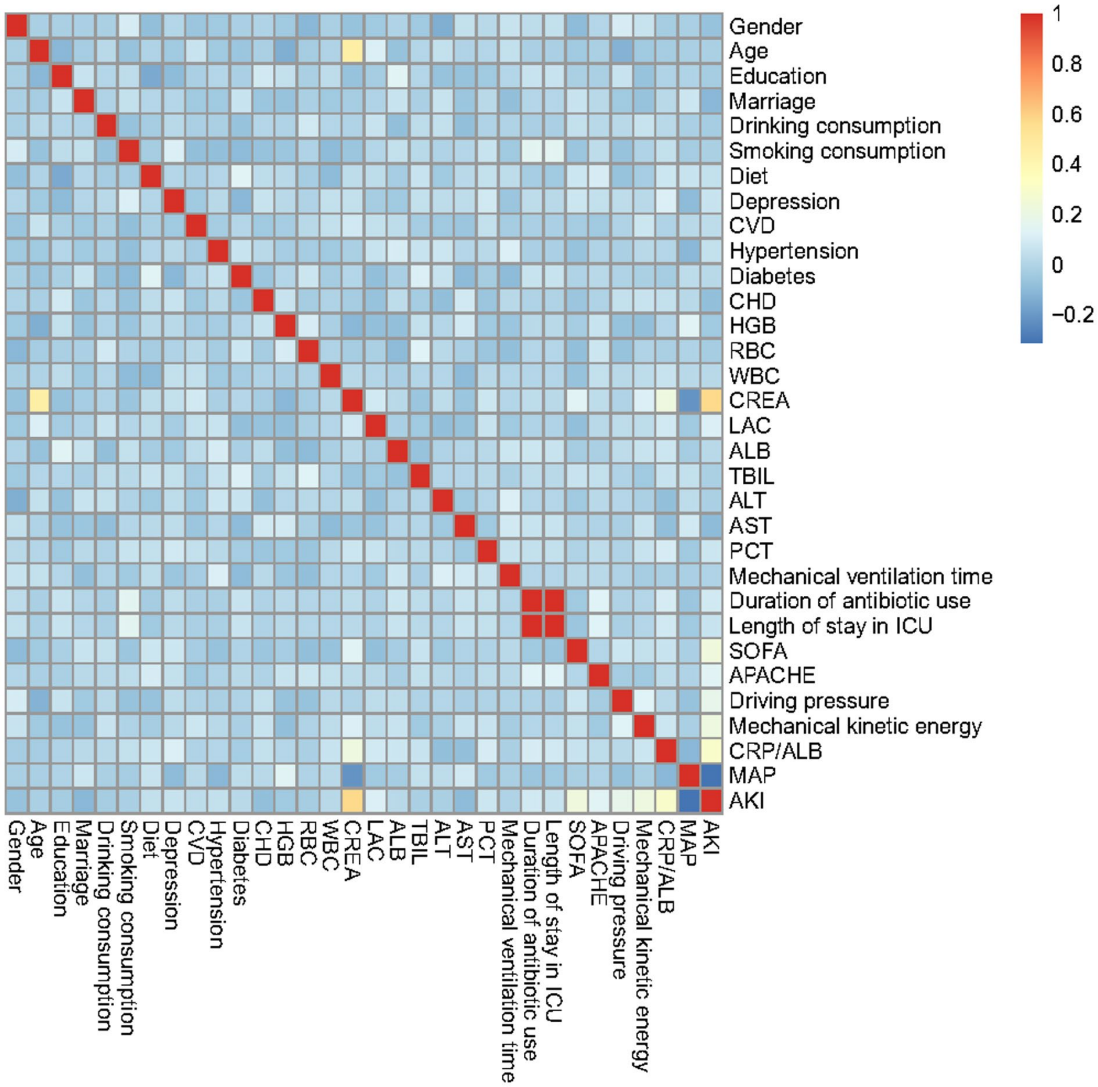
Heatmap of the correlation matrix of characteristics in the training dataset.

Correlation analysis was conducted on the predicted features in the training dataset based on the Pearson method, including gender, age, education, marital status, drinking consumption, and smoking.

consumption, diet, depression, CVD, hypertension, diabetes, CHD, HGB, RBC, WBC, CREA, LAC, ALB, TBIL, ALT, AST, PCT, mechanical ventilation time, duration of antibiotic use, length of stay in ICU, SOFA, APACHE II, driving pressure, mechanical kinetic energy, CRP/ALB, MAP, and AKI, as depicted in Figure 1. In the relevant heatmap, blue and red boxes represent negative and positive correlations, respectively. For example, the duration of antibiotic use is significantly positively correlated with ICU stay, while CREA is negatively correlated with MAP.

### Univariate and multivariate logistic analysis of SPRMV risk factors

In our univariate logistic regression analysis, a total of 9 potential risk factors of SPRMV in the training dataset showed statistically significant results: marriage (unmarried vs. divorced), CREA, LAC, SOFA, APACHE II, driving pressure, mechanical kinetic energy, CRP/ALB, and MAP (*p* < 0.05), as shown in Table 2. Variables analyzed by the univariate logistic regression method were entered into the multivariate logistic regression model based on clinical expertise combined with statistical significance.

**Table 2:** The univariate logistic analysis of common risk factors for SPRMV.

Characteristics	OR (95%CI)	*p*-value
Gender	0.724 (0.462–1.129)	0.157
Age	0.995 (0.967–1.023)	0.714
Education		
Middle school vs. Less than primary school	1.056 (0.624–1.789)	0.839
Upper college vs. Less than primary school	0.852 (0.509–1.425)	0.542
Marriage		
Unmarried vs. Married	0.753 (0.452–1.249)	0.273
Unmarried vs. Divorced	0.574 (0.333–0.987)	0.045
Drinking consumption	0.883 (0.576–1.353)	0.568
Smoking consumption	0.919 (0.600–1.407)	0.698
Diet	1.199 (0.783–1.839)	0.404
Depression	1.254 (0.819–1.927)	0.299
CVD	1.174 (0.767–1.800)	0.460
Hypertension	1.219 (0.796–1.871)	0.364
Diabetes	1.120 (0.732–1.715)	0.603
CHD	0.698 (0.455–1.069)	0.099
HGB, g/L	0.991 (0.971–1.011)	0.368
RBC, 10^12^/L	0.836 (0.469–1.486)	0.542
WBC, 109/L	1.031 (0.901–1.181)	0.652
CREA, μmol/L	1.021 (1.017–1.027)	<0.001
LAC, mmol/L	1.826 (1.063–3.166)	0.030
ALB, g/L	1.098 (0.655–1.844)	0.722
TBIL, μmol/L	0.994 (0.951–1.040)	0.809
ALT, U/L	0.995 (0.972–1.018)	0.661
AST, U/L	0.965 (0.927–1.005)	0.084
PCT, ng/L	1.020 (0.993–1.055)	0.194
Mechanical ventilation time, h	1.000 (0.998–1.002)	0.860
Duration of antibiotic use, h	1.017 (0.997–1.038)	0.100
Length of stay in ICU, days	1.011 (0.992–1.032)	0.266
SOFA	1.094 (1.050–1.142)	<0.001
APACHE II	1.041 (1.010–1.074)	0.009
Driving pressure, mmHg	1.042 (1.016–1.068)	0.001
Mechanical kinetic energy, J	1.046 (1.024–1.069)	<0.001
CRP/ALB	3.144 (2.109–4.780)	<0.001
MAP, mmHg	0.963 (0.950–0.975)	<0.001

Multivariate logistic regression analysis further revealed that CREA (OR = 1.002, 95%CI = 1.002–1.003), SOFA (OR = 1.013, 95%CI = 1.006–1.021), APACHE II (OR = 1.009, 95%CI = 1.004–1.014), driving pressure (OR = 1.007, 95%CI = 1.003–1.012), mechanical kinetic energy (OR = 1.006, 95%CI = 1.002–1.010), CRP/ALB (OR = 1.141, 95%CI = 1.068–1.219) and MAP (OR = 0.995, 95%CI = 0.993–0.997) are independent risk factors of SPRMV (*p* < 0.05) in the elderly patients with AKI, as presented in Table 3. According to the results of multivariate logistic regression analysis, the Odds Ratios (ORs) for continuous variables like CREA (OR = 1.002) and MAP (OR = 0.995) are very close to 1. While statistically significant, their clinical interpretation is challenging. Hence, we presented the OR for a clinically relevant increment (e.g., OR for a 50-unit increase in CREA by dividing the original data by 50, or a 10-unit decrease in MAP by multiplying the original data by 10) to make the results more interpretable for clinicians, as shown in Supplementary Table S5. As depicted in Supplementary Table S5, CREA for a 50-unit increase (OR = 1.117, 95%CI = 1.095–1.138) and MAP for a 10-unit decrease (OR = 1, 95%CI = 0.999–1) are also independent risk factors of SPRMV (*p* < 0.05) in the elderly patients with AKI. However, the regression coefficient for MAP with a 10-unit decrease is zero, and it has no impact on the modified statistical logistic model.

**Table 3:** The multivariate logistic analysis of relevant risk factors for SPRMV.

Characteristics	Beta	Wald	OR (95%CI)	*p*-value
(Intercept)	−0.148	0.710	0.863 (0.612–1.216)	0.400
Marriage				
Unmarried vs. Married	−0.088	3.807	0.916 (0.838–1.000)	0.052
Unmarried vs. Divorced	−0.092	3.560	0.912 (0.830–1.004)	0.060
CREA, μmol/L	0.002	126.893	1.002 (1.002–1.003)	< 0.001
LAC, mmol/L	0.063	1.688	1.065 (0.968–1.171)	0.195
SOFA	0.013	13.409	1.013 (1.006–1.021)	< 0.001
APACHE II	0.009	10.951	1.009 (1.004–1.014)	0.001
Driving pressure, mmHg	0.007	11.576	1.007 (1.003–1.012)	< 0.001
Mechanical kinetic energy, J	0.006	10.691	1.006 (1.002–1.010)	0.001
CRP/ALB	0.132	15.211	1.141 (1.068–1.219)	< 0.001
MAP, mmHg	−0.005	20.119	0.995 (0.993–0.997)	< 0.001

**Figure 2:**
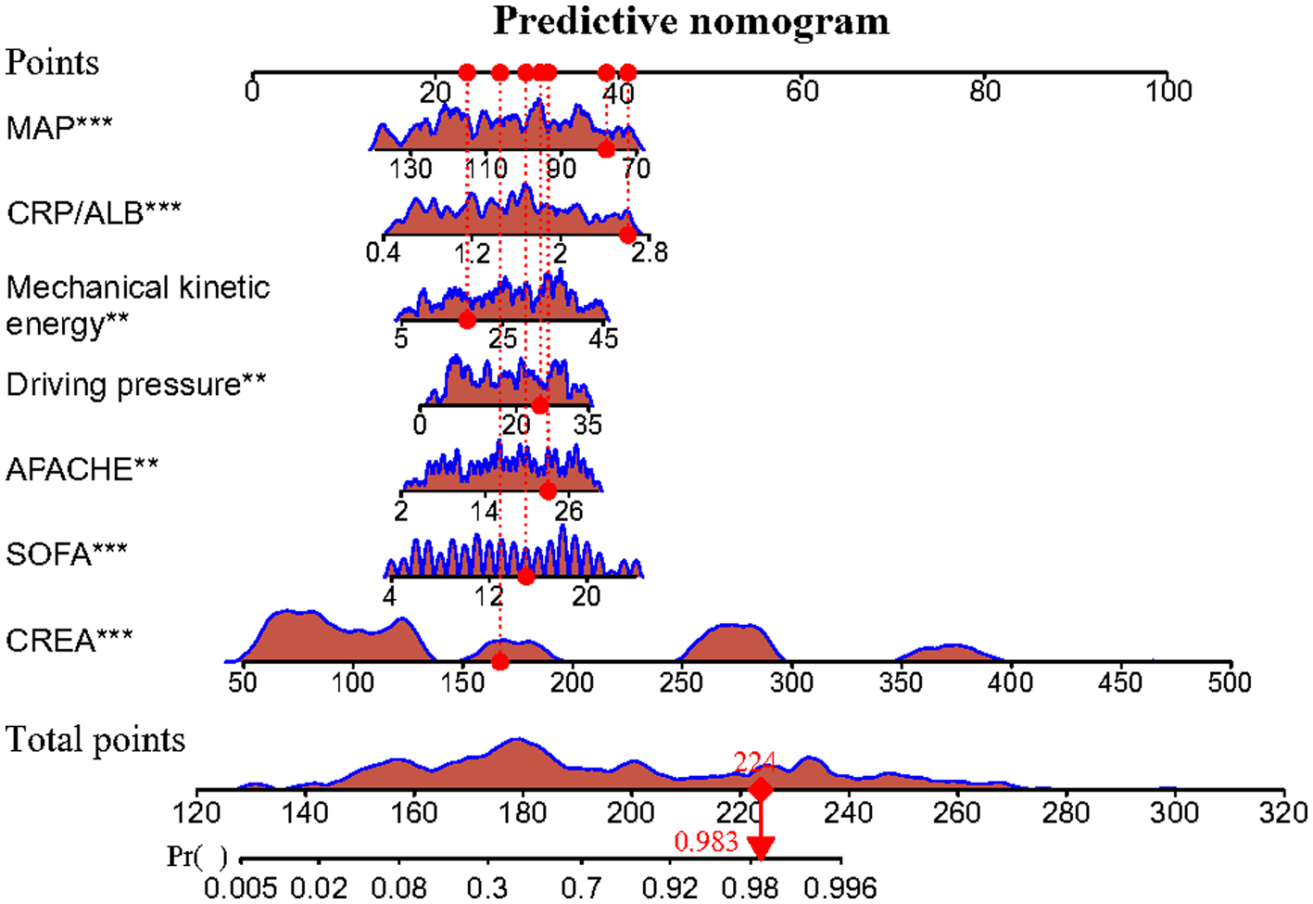
The advanced nomogram for SPRMV patients with AKI.

Meanwhile, the predictive model was presented as an advanced nomogram, which constructed CREA, SOFA, APACHE II, driving pressure, mechanical kinetic energy, CRP/ALB, and MAP based on the aforementioned relevant indicators, as shown in Figure 2. Our advanced nomogram provides a visual representation of the impact of each factor, helping doctors conduct individualized risk evaluations in clinical practice. In addition to the explanation methods in the advanced nomogram in Figure 2, we also gave another example. For example, if an SPRMV patient has CREA (200 μmol/L), SOFA (14), APACHE II (30), driving pressure (20 mmHg), mechanical kinetic energy (25 J), CRP/ALB (2.4), and MAP (140 mmHg), then the SPRMV patient’s corresponding scores would be about 34, 12, 19, 10, 10, 22, and 0, respectively, with a total score of 107. These results would indicate that the estimated probability of SPRMV in the elderly patients with AKI is approximately 91%. Meanwhile, we offered a step-by-step guide in our advanced Figure 2 nomogram. An SPRMV patient with CREA of 170 μmol/L, SOFA of 15, APACHE II of 23, driving pressure of 26 mmHg, mechanical kinetic energy of 18, CRP/ALB of 2.5, and MAP of 81 mmHg, respectively, with a total score of 224. This result would indicate that the estimated probability of SPRMV in the elderly patients with AKI is approximately 98.3%.

### Validation and calibration of predictive model

**Figure 3:**
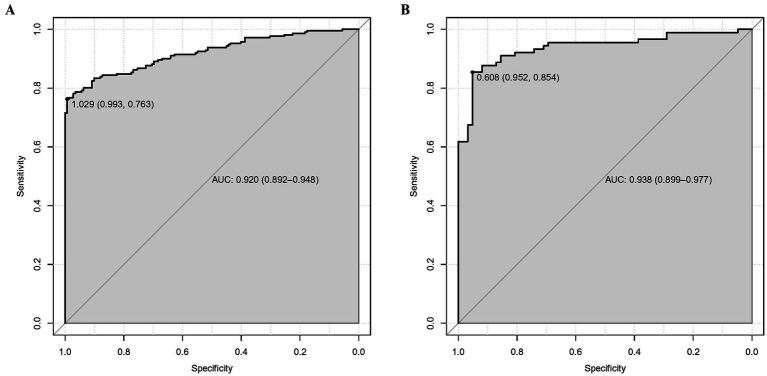
Receiver operating characteristic curve (ROC) of our predictive model. **(A)** Training dataset. **(B)** Validation dataset.

**Figure 4:**
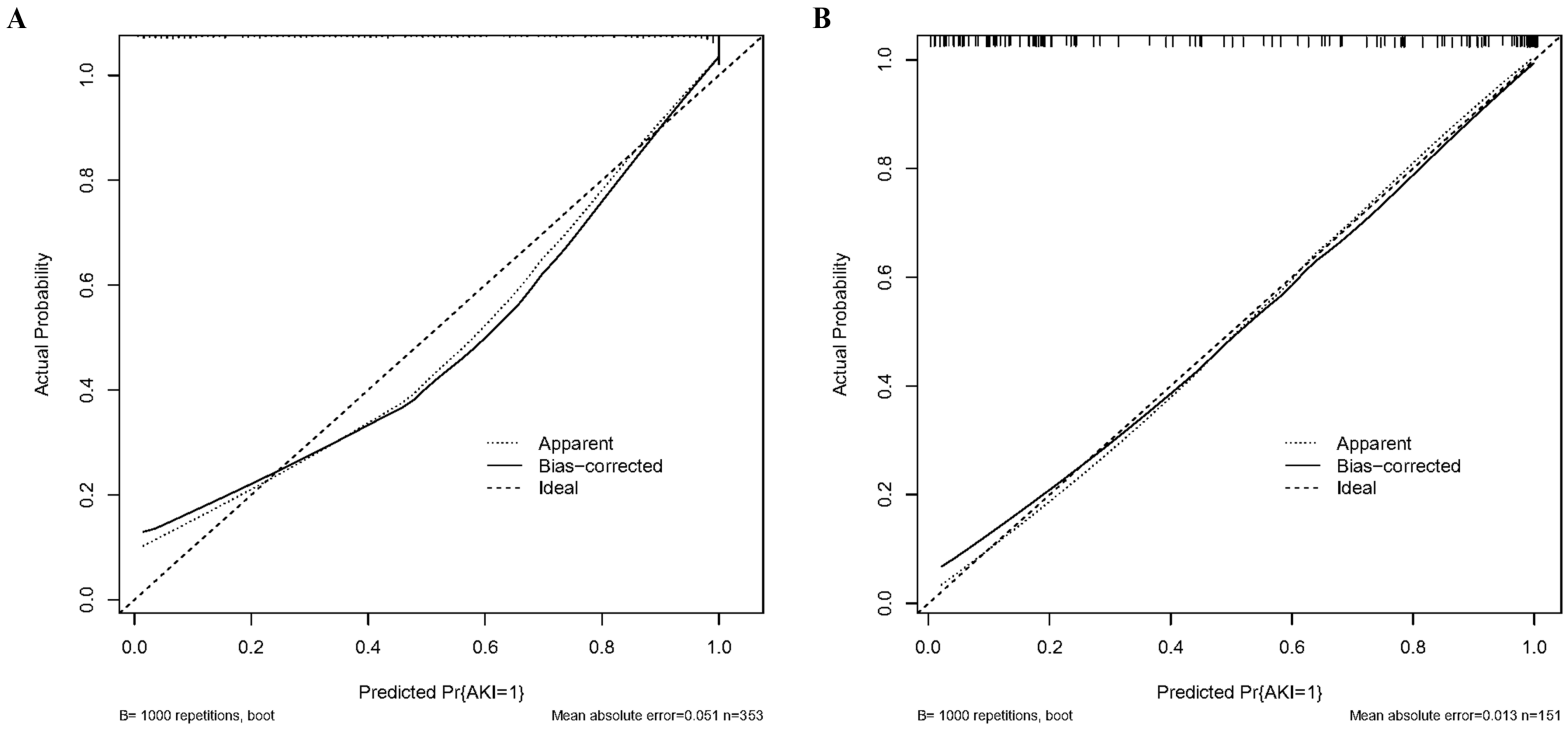
Calibrate the curve of our predictive model. **(A)** Training dataset. **(B)** Validation dataset.

**Figure 5:**
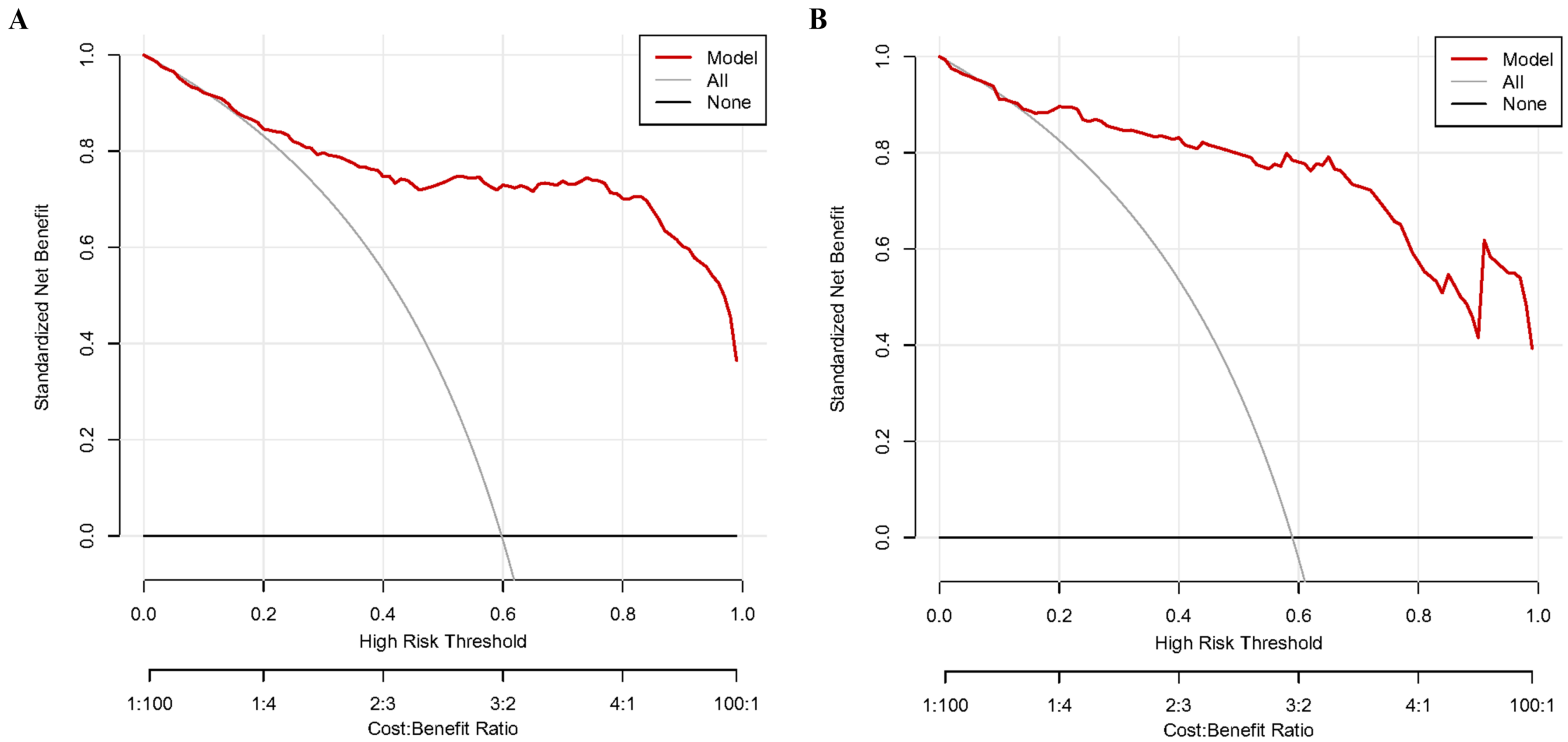
Decision curve analysis (DCA) of our predictive model. **(A)** Training dataset. **(B)** Validation dataset.

As shown in Figures 3A,B, the predictive model generated an AUC of 0.920 (95%: 0.892–0.948) with a specificity of 0.993 and sensitivity of 0.763, and an AUC value of 0.938 (95%CI = 0.899–0.977) with a specificity of 0.952 and sensitivity of 0.854 in the validation dataset. The AUC in validation (0.938) has a higher score than in training (0.920), indicating that our predictive model has a good effect. Calibration curves for the advanced nomogram, based on our multivariate logistic regression analysis across the training and validation datasets, are shown in Figures 4A,B, revealing good agreement between predicted and observed outcomes. In clinical practice, calibration curves are commonly used to evaluate and optimize predictive models, such as the probability of postoperative complications in SPRMV in elderly patients with AKI. By comparing the predicted probability with the actual occurrence probability, doctors can develop more accurate postoperative monitoring and intervention plans. Furthermore, DCA across the training and validation datasets indicates that our predictive model consistently outperforms the two extreme strategies (all treatment and no treatment) in terms of net benefits over a wide range of threshold probabilities, representing its potential clinical capabilities, as shown in Figures 5A,B. According to DCA, doctors can choose the most appropriate intervention threshold based on changes in net income. This helps to avoid excessive or inappropriate interventions and improve the quality of medical decision-making for SPRMV in elderly patients with AKI. The good calibration curve and DCA indicate that our predictive model has good calibration, clinical application, and generalizability.

## Discussion

Our multi-center retrospective analysis developed a clinical predictive model for AKI in elderly patients with SPRMV using a multivariate logistic regression algorithm with clinical data from two ICUs (Hefei Second People’s Hospital and the First Affiliated Hospital of Anhui Medical University), and our predictive model is the first research on AKI in elderly patients with SPRMV. For baseline results, only the baseline HGB differed significantly between the two centers (*p* = 0.047), suggesting good inter-hospital consistency and generalizability of the model. Univariate and multivariate logistic regression analyses identified CREA, SOFA, APACHE II, driving pressure, mechanical kinetic energy, CRP/ALB, and MAP as independent factors associated with AKI in SPRMV elderly patients (*p* < 0.05). AKI remains a frequent and severe complication in this population, largely reflecting the combined impact of systemic inflammation, sepsis, hemodynamic instability, and the physiological burden of mechanical ventilation. These interacting factors jointly impair renal perfusion and function in elderly patients with reduced cardiovascular reserve and comorbidities (25–28).

Among these variables, serum creatinine (CREA) plays a central role as a sensitive marker of renal dysfunction. Elevated CREA reflects compromised glomerular filtration and is strongly associated with AKI incidence and poor outcomes, including prolonged ventilation, extended ICU stay, and increased mortality (29–35). Therefore, continuous monitoring of CREA remains crucial for early AKI detection and for guiding fluid management, nephrotoxin avoidance, and renal-protective interventions in this vulnerable group.

The SOFA score is, therefore, an important prognostic and monitoring tool in elderly patients with SPRMV. It reflects the degree of multi-organ dysfunction in these patients and has very strong predictive value for outcome (36). In these vulnerable patients, severe pneumonia is generally associated with systemic inflammation and sepsis, which may cause failure of organs other than the lungs (37). The SOFA score evaluates organ dysfunction in six systems (respiratory, cardiovascular, liver, coagulation, kidney, and neurological), which is crucial for diagnostic work (38). Therefore, currently, it precisely reflects the overall physiological damage. Elderly people are very sensitive to such damage due to reduced physiological reserves, comorbidities, and immune failure. In these patients, pneumonia often rapidly leads to multiple organ failure, resulting in high SOFA scores (39). A very high SOFA score (≥ 11) upon admission or an increase within 48–72 h in the ICU is a clear independent predictor of extremely high mortality, prolonged ventilation dependence, prolonged ICU stay, and high resource utilization, while a decrease in SOFA score indicates good treatment response (40). The renal dysfunction component in the SOFA score is directly associated with the occurrence of AKI (41). Our study shows that an increase in SOFA score is positively correlated with the incidence of AKI. Therefore, monitoring changes in the SOFA score can not only assess patients’ multiple organ dysfunction, but also provide valuable information for early identification of AKI (42). The application of this indicator can help clinical doctors adjust treatment plans in a timely manner, thereby reducing the risk of further deterioration in kidney function.

The APACHE II score is highly correlated with severe SPRMV in elderly patients. It is a critical illness score based on acute physiological changes, age, and chronic health status upon ICU admission (43). In this high-risk group of elderly patients requiring mechanical ventilation, an increase in APACHE II score is significantly correlated with the hospital’s expected mortality rate (44). Therefore, the APACHE II score is of great significance in evaluating initial severity and prognosis, and in discussing treatment intensity and nursing goals with family members, helping to benchmark ICU performance and stratify clinical research interventions for severe pneumonia (45). Although not the best single predictor, this synthesized APACHE II score provides critical prognostic information: the greater the physiological damage in this group of patients, the more susceptible they are to impact and the lower the expected outcome (46). Meanwhile, some parameters included in APACHE II, such as age, cardiovascular function, and respiratory function, are also closely related to renal function (47). Therefore, the increase in APACHE II score not only reflects the aggravation of multi-organ dysfunction, but may also indicate the risk of AKI occurrence (48).

Driving pressure is the difference between platform pressure and positive end-expiratory pressure (PEEP), a key determinant of ventilation management for SPRMV patients, especially elderly patients (49). Considering the impact of age and disease on lung physiology, this value is the most vulnerable threshold for mechanical ventilation-induced lung injury. Increased driving pressure directly indicates changes in global lung stress and strain during tidal ventilation (50). High driving pressure can cause significant regional stress in rigid lung lesions in elderly patients without requiring excessive ventilation (51). This significantly enhances the mechanism of mechanical ventilation-induced lung injury, including barotrauma, volumetric injury, and atelectasis. Therefore, driving pressure has become a top priority for monitoring and minimizing (52). Our research suggests that driving pressure is closely related to renal dysfunction. The reasons may be related to the following factors: (a) High driving pressure and renal hypoxia: High driving pressure may lead to an exacerbation of systemic inflammatory response, affect renal blood flow perfusion, cause renal hypoxia, and ultimately trigger or worsen AKI (53). Especially in elderly patients, their physiological reserves decrease, and the kidney’s tolerance to hypoxia diminishes (54). (b) Impact of driving pressure on renal fluid balance. High driving pressure may increase the fluid load, thereby increasing the burden on the kidneys (55). Fluid overload can reduce renal blood flow, impair renal excretory function, and exacerbate AKI (56). (c) The necessity of adjusting PEEP: Optimizing PEEP to control driving pressure can not only improve lung ventilation, but also help protect kidney function. Reasonable adjustment of PEEP can reduce lung injury caused by high driving pressure and indirectly lower the risk of AKI (57).

The concept of mechanical kinetic energy, called the energy of moving air, lies at the center of elderly patients with SPRMV. The pneumonia process makes breathing much more inefficient because of reduced lung compliance (making the lungs stiffer) and increased airway resistance due to inflammation and secretions (58). Elderly patients with low muscle strength and reduced physiological reserve are particularly sensitive to this increased energy consumption demand, which can easily lead to respiratory failure (59). Changes in mechanical kinetic energy are also related to acute kidney injury. The possible reasons are as follows: (a) Mechanical kinetic energy and risk of respiratory failure: elderly patients need to spend more mechanical kinetic energy to maintain respiratory function in the process of disease deterioration, which will increase the risk of respiratory failure. Respiratory failure may lead to systemic hypoxia, aggravate kidney injury, and then increase the chance of AKI (60). (b) Impact of gas exchange efficiency: a lack or imbalance of mechanical kinetic energy leads to a decrease in gas exchange efficiency and an increase in complications such as carbon dioxide retention and respiratory acidosis. These metabolic alterations can further compromise renal function, increasing the burden on the kidneys to maintain acid–base homeostasis (61). (c) Ventilator settings and kidney protection: while meeting the demand for mechanical kinetic energy, ventilator parameters should be carefully set to avoid double damage to the lung and kidney (62). Appropriate ventilation mode and setting can help maintain renal function and reduce the incidence of AKI (63). By adjusting the parameters such as reasonable waveform, tidal volume, and respiratory rate, the systemic inflammatory response and potential negative effects on the kidney can be reduced (64). In conclusion, for the ventilation management of elderly patients with SPRMV, monitoring and optimizing driving pressure and mechanical kinetic energy are not only crucial for lung function but also essential for protecting kidney function and reducing the risk of AKI.

CRP/ALB is a very important prognostic biomarker in elderly patients with SPRMV. The ratio reflects two important pathophysiological processes: the level of CRP indicates the degree of systemic inflammatory response syndrome due to infection (65), while the level of ALB reflects nutritional status, hepatic synthetic function, and the degree of an underlying catabolic condition (66). Therefore, high CRP/ALB values reflect a high inflammatory burden associated with physiological deterioration, usually seen in critically ill states (67). Accordingly, increased CRP/ALB at the time of admission or its sustained elevation during an ICU stay is related to increased severity, complications such as acute respiratory distress syndrome, sepsis, and multiple organ dysfunction syndrome, prolonged mechanical ventilation, and longer durations in the ICU and hospitals, with mortality exceedingly high in suspected pneumonia cases among the elderly patients with SPRMV (68). This associate measure, from a clinically available perspective, and its composite marker could stratify risk, predict adverse outcomes, and very likely modulate monitoring and treatment approaches among these clinically fragile and high-risk populations. Increased levels indicate that poorly controlled inflammation and malnutrition/cachexia interact to create a bad prognosis (69). First, the relationship between inflammation and renal function: a high CRP/ALB value in elderly patients with SPRMV not only reflects a high inflammatory burden, but also indicates a potential risk of AKI (67). Chronic low-grade inflammation can induce renal tubular injury, increase the incidence of AKI, and worsen renal function. Therefore, monitoring changes in the CRP/ALB ratio can help clinicians early identify the risk of kidney injury, and then carry out corresponding intervention (70). Second, the impact of nutritional status: a reduction in ALB levels is usually associated with malnutrition, which is an important promoting factor of renal dysfunction. The decrease in ALB levels in elderly patients indicates that albumin synthesis *in vivo* is inhibited, a finding that is significantly correlated with the decline in renal regeneration ability. Therefore, paying attention to the CRP/ALB ratio not only helps monitor the inflammatory state but also reflects the nutritional status of patients, which is related to the risk of AKI (71). Third, the necessity of a comprehensive intervention strategy: combined with the monitoring of the CRP/ALB ratio, a comprehensive intervention strategy should be adopted for elderly patients with SPRMV to reduce the risk of AKI. This includes improving nutritional status, controlling the inflammatory response, and implementing appropriate fluid management to ultimately improve the prognosis of patients (11).

In elderly patients with SPRMV, adequate MAP must be maintained at all times. These patients have a series of high-risk factors for hemodynamic instability: pre-existing cardiovascular disease, low vascular compliance, and systemic inflammatory response caused by severe pneumonia (72). In this context, mechanical ventilation, especially positive-pressure mechanical ventilation, may reduce venous return and cardiac output, thereby exerting complex effects on mechanisms of blood pressure regulation (73). In general, any MAP level above a certain threshold (at least 65 mmHg) will ensure that the perfusion pressure to important organs, especially the kidney and brain, reaches the minimum standard (74). Insufficient MAP will weaken the tissue oxygen and nutrient supply due to infection, further worsen organ dysfunction, and increase mortality. First, the effect of MAP on renal perfusion: the kidney is highly sensitive to blood flow, and a lack of MAP directly affects the renal perfusion pressure, leading to renal hypoxia and AKI. Therefore, maintaining appropriate MAP is an important measure to protect renal function and reduce the risk of AKI (31). Second, the key to fluid management: in the context of mechanical ventilation, a precise fluid resuscitation strategy is needed to ensure effective hemodynamic support and maintain MAP level, thereby maintaining renal perfusion and function. This implies that during fluid resuscitation, hemodynamics needs to be continuously monitored to ensure patients receive the best support (75). Third, monitoring and intervention for critically ill patients: given the urgent need for appropriate MAP, continuous MAP monitoring and necessary interventions are essential. Through standardized monitoring and targeted intervention, it can maintain organ perfusion and improve prognosis in the setting of severe infection and mechanical ventilation, especially by actively protecting renal function (76). In conclusion, the CRP/ALB ratio and MAP not only play an important role in evaluating the severity and prognosis of elderly patients with SPRMV but are also associated with the occurrence of AKI. Effective monitoring and management of these indicators can guide clinical decision-making and improve patient outcomes and quality of life.

Moreover, our multivariate logistic regression model revealed that CREA, SOFA, APACHE II, driving pressure, mechanical kinetic energy, CRP/ALB ratio, and MAP will be independent risk factors for AKI among SPRMV patients. Even after analysis removes the influence of confounding factors through intervention, it becomes relatively straightforward to view an important determinant of AKI as a complex, multivariate phenomenon in this critically ill population. One variable might act through one or more important interactions with others, thus modifying the net impact on renal outcomes that any of the measured variables could independently be expected to have. For the interaction of hemodynamics and inflammation, a usually low MAP may interact unfavorably with a high CRP/ALB ratio, an inflammatory marker and indicator of systemic oxidative stress (77). Inflammation could interfere with renal autoregulation, thus increasing the kidney’s vulnerability to even brief episodes of relative hypotension. The nephrotoxic effects of inflammation might be lessened by strict hemodynamic management (78). And when it comes to ventilatory-induced kidney injury and severity of illness, the association between the determinants of ventilator-induced lung injury and AKI may be modified by a patient’s pre-existing physiologic reserve. High driving pressure or mechanical kinetic energy deteriorates renal venous congestion and cardiac output; therefore, these parameters would be magnified in those patients with high APACHE II or SOFA scores with greater baseline organ dysfunction and hemodynamic instability (79, 80). Besides, our predictive model can be integrated into clinical practice. Based on our multivariate logistic regression, we outline a possible path for clinical integration, such as embedding the model in the electronic health record (EHR) as a decision-support tool to flag high-risk patients for physician review. Furthermore, we note that effective facilitators include an interface that is easy to use and fits with current clinical practices, whereas significant barriers include concerns over data privacy, regulatory approval (e.g., FDA clearance), and the need for clinical validation. We believe that this addition greatly enhances the practical relevance of our study.

Furthermore, there are certain limitations in our multi-center retrospective analysis. First of all, as a multi-center study, the cohort may not represent broader demographic or geographic populations, and the exclusion of patients with chronic renal disease stage 4–5 will affect pre-existing renal dysfunction, limiting model generalizability. Meanwhile, unmeasured confounding factors may also affect the predictive model, and further research will be carried out in the future. Furthermore, our model does not explicitly mention whether a power calculation was conducted to determine the appropriate sample size. The Events per Variable (EPV) metric is intended to estimate the sample size of a predictive model in the future (26, 81). Second, univariate and multivariate logistic regression are prevailing methods used in our analysis. Furthermore, machine learning algorithms can be used to establish new predictive models with better performance (82). Third, there may be biases in the recording of more effective features of our predictive model, including short follow-up time, serological and histological variability, and histopathological grading, which are influenced by inter-laboratory variability and inter-observer differences. In the future, the standardization and linkage of electronic health records will promote the standardization and interoperability of EHR data across different medical institutions and form a regional or national health information database. This will greatly expand the sample size, improve representativeness, and reduce selection bias. And integrating multi-source data will combine traditional medical data with medical insurance settlement data, death registration data, environmental monitoring data, and even health data generated by wearable devices. This data fusion can more comprehensively describe the individual’s exposure history and health status and provide a new research perspective.

## Conclusion

In our multi-center retrospective analysis, we found a relationship between several independent factors (CREA, SOFA, APACHE II, driving pressure, mechanical kinetic energy, CRP/ALB, and MAP) and AKI in SPRMV patients and developed a predictive model to evaluate the clinical diagnosis of SPRMV.

## Data Availability

The original contributions presented in the study are included in the article/Supplementary material, further inquiries can be directed to the corresponding author.
